# Haematological and Oncological Training Therapy With Stationary Strength and Cardio Machines (HOT) in Routine Cancer Care: A 3‐Year Real‐World Evaluation of Acceptance, Feasibility, Safety, and Effects

**DOI:** 10.1002/cam4.72013

**Published:** 2026-06-12

**Authors:** Sabine Felser, Maya Engel, Christina Grosse‐Thie, Brigitte Kragl, Larissa Henze, Imke Albrecht, Hans Lampe, Susanne Fischer, Ulrich Langenkamp, Christian Junghanss

**Affiliations:** ^1^ Department of Internal Medicine, Clinic for Hematology, Hemostaseology, Oncology, Stem Cell Therapy and Palliative Care Rostock University Medical Center Rostock Germany; ^2^ Hematology and Oncology Practice Rostock Germany; ^3^ Oncology Centre of the Rostock University Medical Centre Rostock Germany; ^4^ Department of Internal Medicine II—Hematology, Oncology and Palliative Medicine Asklepios Hospital Harz Goslar Germany; ^5^ Department of Physiotherapy Rostock University Medical Center Rostock Germany; ^6^ Mittelstand‐Digital Zentrum Rostock, Stabsstelle Ärztlicher Vorstand Rostock University Medical Center Rostock Germany

**Keywords:** cancer, chemotherapy‐induced peripheral neuropathy (CIPN), EORTC QLQ‐C30, exercise therapy, fatigue, health‐related quality of life (HRQoL)

## Abstract

**Background:**

Exercise improves physical function and health‐related quality of life (HRQoL) in patients with cancer; however, nationwide structured programmes remain limited. A hospital in Germany implemented a donation‐funded Haematological and Oncological Training Therapy with Stationary Strength and Cardio Machines (HOT), and a 3‐year real‐world data analysis aimed to evaluate its acceptance, feasibility, safety, and effects.

**Methods:**

HOT comprised 24 sessions of combined resistance and aerobic training, complemented by a sensorimotor module for patients with chemotherapy‐induced peripheral neuropathy (CIPN), with a target frequency of two sessions per week. Patients with all cancer types and treatment phases were eligible. The study evaluated acceptance (e.g., participants' clinical and sociodemographic characteristics, reasons for premature discontinuation), feasibility (e.g., completion rate, training density), safety (adverse events), and effects (changes in strength [1‐repetition maximum, 1‐RM], cardiopulmonary performance [maximum power on a cycle ergometer], and patient‐reported outcomes [HRQoL, fatigue, CIPN]) within a quasi‐experimental pre–post design.

**Results:**

Between April 2021 and March 2024, 70 patients (60% female; mean age, 63 ± 12 years; range, 31–91 years) participated; 61% had solid tumours and 39% haematological malignancies, and 57% were undergoing active cancer treatment. Most participants (94%) had a history of sports participation. A total of 53 participants (76%) completed HOT, while 17 (24%) discontinued prematurely, mainly for health‐related reasons. The training density among completers was 1.6 sessions per week. One adverse event occurred during a 1‐RM test. Significant improvements were observed in strength and cardiopulmonary performance (*p* < 0.05), as well as in global HRQoL (*p* < 0.001), fatigue (*p* < 0.001), and CIPN (*p* = 0.003).

**Conclusion:**

HOT appeared to primarily attract sports‐experienced patients, with haematological malignancies overrepresented. HOT proved to be safe and feasible across diverse cancer entities and treatment phases and was associated with improvement in physical function and HRQoL. The participants' high baseline motivation for exercise may limit generalisability.

## 1 Background

Strong evidence shows that structured exercise can improve physical function and health‐related quality of life (HRQoL) in patients with cancer. Randomised controlled trials, systematic reviews, and meta‐analyses demonstrate benefits for physical performance [1, 2] reduction in symptoms such as fatigue [3, 4] and chemotherapy‐induced peripheral neuropathy (CIPN) [5, 6], and improvements in HRQoL [1, 7]. Exercise during neoadjuvant, primary, and adjuvant treatment may enhance cancer therapy efficacy [8], and physical activity after diagnosis can improve survival for certain cancers [9, 10]. These benefits are thought to be mediated by a combination of systemic and cellular mechanisms, including anti‐inflammatory effects, improved immune function, and modulation of tumour‐related pathways [11, 12]. Consequently, major health organisations recommend integrating structured exercise into oncology care [13, 14, 15]. However, implementation varies internationally, depending on healthcare systems and policy frameworks [15].

Quality‐assured oncological exercise therapy before and during treatment is not consistently implemented as part of standard care, including in Germany. In Germany, structured programmes are lacking, and “rehabilitation sport”, reimbursed by statutory health or pension insurance, is restricted to cancer survivors and excludes stationary exercise equipment [16]. Inpatient or outpatient rehabilitation is usually available only after treatment completion and lasts for 3 weeks, combining multimodal services such as health education, psychotherapy, nutrition, and exercise. A noteworthy model is the Oncological Training and Exercise Therapy (OTT) programme, developed at the Cologne University Hospital in 2010. This programme translates current evidence into practice through a structured programme of 24 sessions on stationary equipment [17]. However, OTT is not reimbursed by statutory health insurance. This is one reason why only a few locations in Germany offer such training programmes. Stationary exercise equipment supports current recommendations—three sessions of moderate‐intensity aerobic exercise and two sessions of resistance training per week [13]. Compared with free weights or bodyweight exercises, these devices offer guided movements for greater safety, targeted muscle group activation, adjustable resistance for individual progression, and quick exercise transitions [18, 19, 20]. These advantages are particularly relevant for older adults, patients with low muscle strength, and individuals undergoing active treatment. Although feasibility has been shown in controlled trials, real‐world evidence remains scarce, especially regarding which patients voluntarily use such programmes and how they respond.

In Mecklenburg–Western Pomerania (M‐V), Germany's least densely populated state (70 inhabitants per km^2^) [21], no programmes comparable to OTT exist. In Rostock (the largest city in M‐V, with 210,000 inhabitants) and its surrounding district, approximately 3000 new cancer cases are diagnosed annually (M‐V Cancer Registry). To provide access to structured exercise, a voluntary, donation‐funded programme—Haematological and Oncological Training Therapy with Stationary Strength and Cardio Machines (HOT)—was launched at the University Medical Center Rostock (UMR), based on the OTT concept. HOT is open to all cancer types and treatment phases.

In this observational study, we evaluated the programme's acceptance, feasibility, safety, and effects over a 3‐year period, and assessed whether the eligibility criteria and frequency, intensity, time, and type (FITT) criteria support safe and effective training in a heterogeneous patient population.

## 2 Methods

### 2.1 Study Design

This observational study followed a quasi‐experimental, one‐group pre–post design.

### 2.2 Participants, Eligibility Criteria, and Settings

The study was conducted at the Clinic for Hematology, Hemostaseology, Oncology, Stem Cell Therapy and Palliative Care at the UMR. The first patient was enrolled on 15 April 2021.

Eligible participants were ≥ 18 years old with a diagnosis of malignant neoplasms (ICD codes C00*–C97*) or neoplasms of uncertain or unknown behaviour of the haematopoietic or lymphatic system (D47), sufficient German language skills, and written medical clearance for exercise by a haematology/oncology specialist. Absolute and relative contraindications are listed in Table 1. Participants with absolute contraindications were excluded but could be re‐screened at a later date. In cases of relative contraindications, participation was permitted after appropriate medical management (e.g., correction of electrolyte imbalances). For patients with osteoarthritis, rheumatologic diseases, or neutropenia, the training programme and performance diagnostics were adapted in consultation with the treating physician. The HOT program is primarily intended for patients undergoing medical treatment (e.g., systemic therapy, radiotherapy, pre‐ or post‐surgery), those with chronic or palliative disease, as well as patients in aftercare (defined as a cancer diagnosis within the past 24 months). For patients in follow‐up care, participation was scheduled to avoid overlap with inpatient rehabilitation. Rehabilitation had to be completed prior to study enrolment or was considered as an exclusion criterion if it occurred during the study period. In exceptional cases, patients in follow‐up care more than 24 months post‐diagnosis were included in the HOT program, particularly if they presented with persistent disease‐ or treatment‐related side effects (e.g., fatigue, CIPN, or reduced physical performance).

**TABLE 1:** Inclusion criteria and contraindications for haematological and oncological training therapy with stationary strength and cardio machines.

**Inclusion criteria**
Age ≥ 18 years Any type of cancer diagnosis ICD: C00*–C97* and D47 Sufficient knowledge of German to understand the instructions Medical clearance to perform sports (issued by a specialist in haematology and oncology)
**Absolute contraindications**
Patients who fulfill at least one of the following criteria will not be included: Non‐consenting patients Acute infectious diseases Fresh wounds or incomplete wound healing after surgery Myocardial infarction within the last 4 weeks Unstable angina pectoris Clinically relevant heart failure (NYHA III and IV) Higher‐grade valvular vitia Uncontrolled cardiac arrhythmias Chronic obstructive pulmonary disease (GOLD III and IV) Thrombosis/pulmonary embolism New severe pain symptoms Haemoglobin value < 4.8 mmol/L Platelets < 20 Gpt/l
**Relative contraindications**
Osteoarthritis (depending on severity, exercise reduction if necessary) Rheumatological disease (depending on the degree of activity) Hypertension (if not stably adjusted) Diabetes mellitus (if insufficiently controlled) Electrolyte imbalances (if cannot be compensated) Hypercalcaemia > 2.6 mmol/L Hypokalaemia < 3.2 mmol/L Hyperkalaemia > 5 mmol/L Hyponatraemia < 130 mmol/L Hypernatraemia > 148 mmol/L Bronchial asthma (in acute attacks) Acute allergies Neutropenia (no group training possible) Peripheral arterial occlusive disease

The study was conducted in accordance with the Declaration of Helsinki [22] and approved by the Ethics Committee of the University of Rostock (A2020‐0211). The study was registered in the German Clinical Trials Register (DRKS00023912), and all participants provided written informed consent.

### 2.3 FITT Criteria

The HOT programme was adapted from the OTT programme (Cologne University Hospital) [23] and comprised 24 sessions of combined resistance and aerobic training using stationary strength and cardio machines. Training was conducted twice weekly at the Olympic Training Center M–V in Rostock under the supervision of OTT‐certified therapists (staff‐to‐patient ratio, 1:5).

In the basic training (BT), resistance training included six exercises (leg extension, leg curl, abdominal crunch, back extension, seated rowing, and chest press), performed on computerised strength machines (milon Q; milon industries GmbH, Emersacker, Germany). The exercises were performed concentrically, and each device provided interactive live feedback on movement execution (Additional File 1). Equipment settings were individualised, and training load was based on the 1‐repetition maximum (1‐RM) and adjusted as needed. Intensity was regulated using the 15‐point Borg scale (6 = very, very easy; 20 = maximal exertion) [24], targeting moderate to vigorous levels (Borg 14–16). Aerobic training consisted of 4‐min cycling intervals at moderate intensity (Borg 12–14).

**FIGURE 1:**
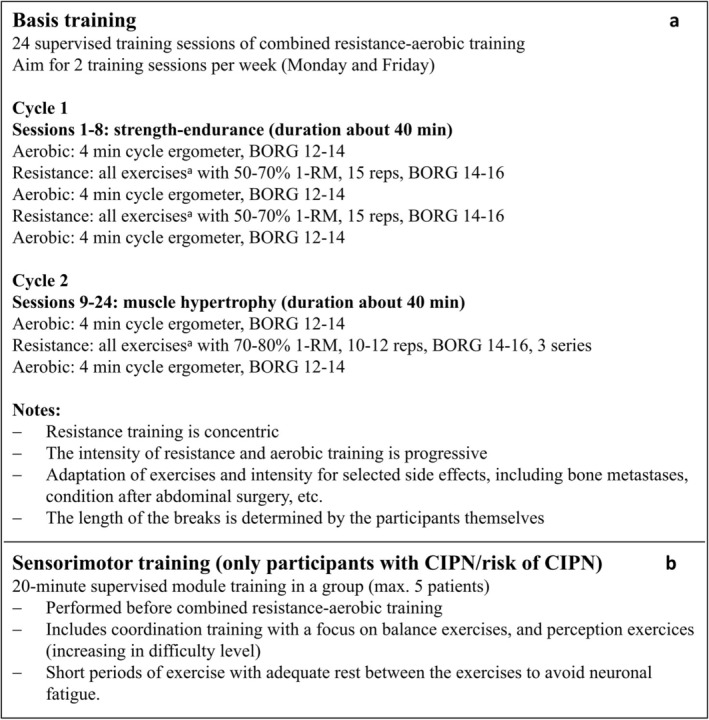
FITT criteria for basic training (a) and sensorimotor training (b). min, minutes; reps, repetitions; ^a^includes six exercises: leg extension, leg curl, abdominal crunch, back extension, seated rowing, chest press.

In the BT, sessions 1–8 emphasised strength–endurance and movement efficiency, while sessions 9–24 focused on hypertrophy. The FITT criteria are shown in Figure 1a. If contraindications limited specific exercises, alternative modalities (e.g., resistance bands, bodyweight exercises, treadmill walking) were applied (“adapted BT”).

Participants with or at risk for CIPN additionally performed 20 min of group‐based sensorimotor training (SMT) before each session, focusing on coordination, balance, and perception (Figure 1b).

### 2.4 Procedure

The HOT programme was offered free of charge because it was funded through donations. Participation was voluntary, did not require a physician's referral, and was open to patients with all cancer types and treatment phases, in case they met the eligibility criteria described above. Recruitment was conducted via in‐hospital promotion, flyers, collaborating practices, the hospital website, and word of mouth. At programme launch in April 2021, capacity was 5 slots (1 group); since summer 2022, this has doubled to 10 slots (2 groups).

Interested patients contacted the clinic and received information on procedures and training content from a certified therapist. If slots were available, written informed consent for study participation was obtained; otherwise, patients were placed on a waiting list.

Patient‐reported outcomes (PROs) were digitally recorded, and a medical clearance examination was conducted. Based on the clinical data collected during this examination, participants were assigned to one of the following groups: “(adapted) BT” (Group 1) or “(adapted) BT + SMT” (Group 2). If a training programme significantly different from BT was required, such as for patients with multiple metastases, participants were assigned to “no BT (+ SMT)” (Group 3).

If the physician had safety concerns regarding the execution of the physical performance diagnostics, such as the determination of the 1‐RM, these concerns were discussed with the therapists. Within 10 days of obtaining medical clearance, participants were introduced to the strength machines and underwent the initial physical performance diagnostics. After this, they began the HOT programme. Following the 24‐session HOT programme, post‐intervention physical performance diagnostics and PRO collection were performed, and results were summarised in a personalised assessment report. In a final consultation with a haematologist/oncologist, the results were discussed and the report provided (Additional File 2).

No modifications to the HOT programme were made during the 3‐year observation period. All seven therapists involved were physiotherapists or exercise scientists. Prior to delivering the intervention and conducting physical performance diagnostics, all staff were trained according to the study protocol and supervised by certified OTT therapists during the initial sessions to ensure standardization. For SMT, a standardized exercise catalogue was used by all therapists. Training on milon devices was electronically guided and monitored. In addition, monthly team meetings were held to ensure consistency in programme delivery.

### 2.5 Data Collection

The data collection schedule is shown in Table 2.

**TABLE 2:** Data collection schedule.

Assessments	Pre	Post
*Physician*		
Disease‐specific data (questionnaire) cancer type, entity, therapy intention, previous treatments, current therapy phase, current therapies, risk of CIPN, side effects/bone metastases, and comorbidities	✓	
Satisfaction of the participants		✓
*Physical performance diagnostic*		
Body composition (BIA) fat mass [kg], body fat [%], skeletal muscle mass [kg] Body Mass Index [kg/m^2^]	✓	✓
1‐Repetition maximum [kg]^a^ leg extension, leg flexion, abdominal press, back extension, row, chest press	✓	✓
Cardiopulmonary performance (maximal exercise test on a cycle ergometer) maximum power [W], relative maximum power [W/kg BW], rate of perceived exertion [Borg scale], exercise‐induced leg muscle pain [CR‐10], maximum heart rate [bpm]	✓	✓
*Patient*		
Sociodemographic data (initial questionnaire) age, sex, family status, professional status, sports history	✓	
Quality of life (EORTC QLQ‐C30)	✓	✓
Fatigue (MFI‐20)	✓	✓
Neuropathy (FACT/GOG‐Ntx‐13 subscale)	(✓)	(✓)
Physical activity (GSLTPAQ)	✓	✓
*Therapist*		
Training group assignment	✓	
Adverse events (protocol)	Accompanying, separately for training and physical performance diagnostics in case of premature discontinuation	
Reasons and timing of premature discontinuation		
*Research team*		
Dates of the 1st and 24th training sessions		✓

*Note:* (✓) Recording only if a chemotherapy‐induced peripheral neuropathy is present before the start of training.

Abbreviations: BIA, bioimpedance analysis; bpm, beats per minute; BW, body weight; CIPN, chemotherapy‐induced peripheral neuropathy; CR‐10, Category‐Ratio‐10 scale (0 = no pain at all, 10 = extremely intense pain); EORTC QLQ‐C30, Quality of Life questionnaire of cancer patients of European Organization for Research and Treatment of Cancer; FACT/GOG‐NTX‐13, Functional Assessment of Cancer Therapy/Gynecologic Oncology Group—Neurotoxicity 13 Item Version; GSLTPAQ, Godin‐Shephard Leisure‐Time Physical Activity Questionnaire; MFI‐20, Multidimensional Fatigue Inventory.

^a^ Complete test implementation only in basic training; individual tests may be omitted in adapted basic training.

*Disease‐specific data* (e.g., cancer type, entity, therapy intention, previous treatments, current therapy phase, current therapies, risk of CIPN, side effects/bone metastases, and comorbidities) were recorded once by the treating or recruiting physicians at the time of patient inclusion.

Before and after completion of the 24 training sessions, physical performance diagnostics were conducted and PROs were recorded digitally. The assessment included the following measures.

#### 2.5.1 Physical Performance Diagnostics

Physical performance assessments were conducted before the first (pre) and after the 24th (post) training session, with no more than 10 days between medical clearance and the pre‐diagnostic assessment. Efforts were made to ensure that the same therapist conducted both the pre‐ and post‐tests for each participant, and that the order of the tests remained constant. The evaluation consisted of the following measures.

- *Body composition* (fat mass [kg], body fat [%], and skeletal muscle mass [kg]) was determined using a mobile bioimpedance analyser (seca mBCA 525; seca GmbH, Hamburg, Germany). Measurements were conducted according to the user manual. Body mass index was calculated as body weight (kg) divided by height (m^2^). Percentage changes in fat and muscle mass were also calculated.
- *1‐RM* was determined on computerised strength training devices of the milon Q type (milon industries GmbH), including leg extension, leg curl, abdominal crunch, back extension, seated rowing, and chest press. The maximum dynamic strength of each muscle group was quantified under standardised conditions using the isokinetic (dynamometric) mode at a constant angular velocity. The device guided the movements, and the range of motion was adjusted individually. Before the pre‐test, sufficient repetitions were performed to learn the movement pattern, which also served as a warm‐up. In the post‐test, a 5‐min general warm‐up on the cycle ergometer was followed by five to seven repetitions with submaximal weights to warm up the specific muscle group. After a short break, at least two 1‐RM measurements were conducted, with at least 30 s of rest between them. If the second measured value was higher than the first, additional measurements were taken until the 1‐RM plateaued or decreased. For safety reasons, the 1‐RM of individual muscle groups was not measured if contraindications (e.g., bone metastases, recent abdominal surgery) were present. Percentage changes in 1‐RM were calculated for each muscle group.
- *Cardiopulmonary performance* was determined using a maximal exercise test on a cycle ergometer (SportPlus, SP‐SRP‐3000; Latupo GmbH, Hamburg, Germany) in accordance with the method reported by Löllgen and Leyk [25]. The test started at 20 watts and increased by 10 watts each minute (quasi‐ramp test). Pedalling speed was maintained at 60–80 rpm (±5 rpm allowed). The test continued until subjective exhaustion or the appearance of termination criteria (e.g., discomfort or dizziness). In the last 10 s of each load stage, heart rate was recorded using a chest strap (Polar H10; Polar Electro Oy, Kempele, Finland), and the rate of perceived exertion (RPE) was measured using the Borg scale [24]. Immediately after test termination, time, heart rate, RPE, and exercise‐induced leg muscle pain were recorded using the Category‐Ratio (CR)‐10 scale (0 = no pain at all, 10 = extremely intense pain) [26]. Maximum power and relative maximum power were calculated based on the time completed during the last stage, and percentage changes from pre‐ to post‐test were calculated.

#### 2.5.2 PROs

d *Sociodemographic data* (age, sex, family status, and professional status) and sports history were collected using an initial questionnaire.
e *HRQoL* was measured using the established Quality of Life Questionnaire from the European Organisation for Research and Treatment of Cancer (EORTC QLQ‐C30, version 3.0) [27, 28]. The validated questionnaire includes a scale for global HRQoL, five functional scales, three symptom scales, and six single‐item symptoms. No threshold values were set in advance for assessing clinically important differences.
f *Fatigue* was assessed using the Multidimensional Fatigue Inventory (MFI‐20) [29], which contains 20 items spanning five dimensions: general fatigue, physical fatigue, reduced activity, reduced motivation, and mental fatigue. The total fatigue score was calculated from all 20 items.
g *CIPN* was evaluated using the Functional Assessment of Cancer Therapy/Gynecologic Oncology Group–Neurotoxicity‐13 (FACT/GOG‐NTX‐13) subscale, which assesses the degree and impact of sensory and motor neurotoxicity symptoms [30]. This test was administered only to participants who already had symptoms of CIPN at inclusion.
h *Physical activity* was assessed using the Godin–Shephard Leisure‐Time Physical Activity Questionnaire (GSLTPAQ) [31], a four‐item self‐administered questionnaire. Three questions gathered information on the frequency of mild, moderate, and strenuous physical activity lasting at least 15 min per session during a typical week. The Leisure Score Index (LSI) was calculated by multiplying the number of activity bouts at each intensity by metabolic equivalents (3, 5, and 9 for mild, moderate, and strenuous, respectively) and summing the results. An LSI of ≥ 24 is defined as physically active, 14–23 as moderately active, and < 14 as insufficiently active.

Absolute changes in scores (Δ‐values) were calculated for the global HRQoL scale and the five functional scales of the EORTC QLQ‐C30, the total fatigue score of the MFI‐20, the neurotoxicity subscale of the FACT/GOG‐NTX‐13, and the LSI.

*Participant training group assignments* were documented by the therapists.

*Adverse events (AEs)* that occurred during training or physical performance diagnostics were recorded by the therapists. A distinction was made between AEs (health‐related incidents leading to temporary impairment of physical function or health that require, at most, medical evaluation but no treatment) and serious AEs (SAEs) (health‐related incidents during training resulting in medium‐ or long‐term impairment of physical function or health and requiring medical treatment). All AEs were documented in a protocol and categorised according to their relation to HOT as “definite”, “possible”, “unlikely”, or “not assessable”. The procedure for handling SAEs was clearly regulated and involved a defined rescue chain. To clarify the relationship between an SAE and HOT, a safety board consisting of an emergency physician and two oncologists was consulted.

*Reasons for and timing of premature discontinuation* were collected by the therapist.

*Intervention duration*, based on the dates of the 1st and 24th sessions recorded in the milon database (electronic chip card system), was divided by 24 to calculate mean training density (sessions/week).

*Participant satisfaction* was assessed during the final consultation with a haematologist/oncologist and dichotomised as yes or no.

### 2.6 Outcome Measures

Endpoints included acceptance, feasibility, safety, and effects of HOT. Acceptance was assessed by determinants of participation (clinical and sociodemographic characteristics), reasons for premature discontinuation, and satisfaction. Feasibility was evaluated by training group assignment, completion rate, and adherence, the latter defined as training density (sessions per week). Safety was monitored via AEs during training and performance testing. Effects were assessed by pre–post changes in physical performance (body composition, 1‐RM, and maximum power on a cycle ergometer) and PROs (HRQoL, fatigue, CIPN, physical activity), with clinical relevance considered where applicable.

The selected established physical performance tests and validated PROs were based on the standardized assessment framework of the OTT program, ensuring comparability with other exercise oncology providers.

### 2.7 Sample Size Calculation

In this real‐world data analysis, an a priori sample size calculation was not conducted because the study utilised existing datasets, with the sample size determined by the availability and completeness of the data. The dataset included 70 participants, who were selected based on predefined eligibility criteria to ensure representativeness of the target population.

### 2.8 Statistical Analysis

Quantitative variables are presented as mean ± standard deviation or, in cases of non‐normal distribution, as median (Q1, Q3), with ranges from minimum to maximum. Qualitative variables are presented as absolute counts (*n*) and relative frequencies (%). Missing data points were not imputed, and all statistical analyses were conducted using a complete‐case approach, including only participants with available data for the respective variables and timepoints. Normality of the data distribution was assessed using the Shapiro–Wilk test. Differences between completers and non‐completers for continuous variables were analysed using the independent *t*‐test or, in cases of non‐normality, the Mann–Whitney *U* test (Monte Carlo, 10,000 samples). For categorical variables, the chi‐squared test or Fisher's exact test was applied, as appropriate.

Changes in physical performance and PROs were analysed using the paired *t*‐test or, in cases of non‐normality, the Wilcoxon signed‐rank test (Monte Carlo, 10,000 samples). Cohen's *d* was calculated to estimate effect sizes (ES), reported as absolute values, with thresholds defined as follows: 0.2 ≤ *d* < 0.5 = small, 0.5 ≤ *d* < 0.8 = medium, ≥ 0.8 = large. Pearson correlations were calculated to assess the association between changes in skeletal muscle mass and changes in strength. Subgroup analyses were conducted, stratified by cancer type (solid vs. haematologic neoplasms), treatment status (undergoing treatment vs. aftercare/watch‐and‐wait), presence of CIPN at baseline, and sex. Group differences were analysed using the exact Mann–Whitney *U* test based on percentage changes or Δ‐values. A two‐sided *p*‐value of < 0.05 was considered statistically significant. All analyses were performed using SPSS (IBM Corp., Armonk, NY, USA).

## 3 Results

**FIGURE 2:**
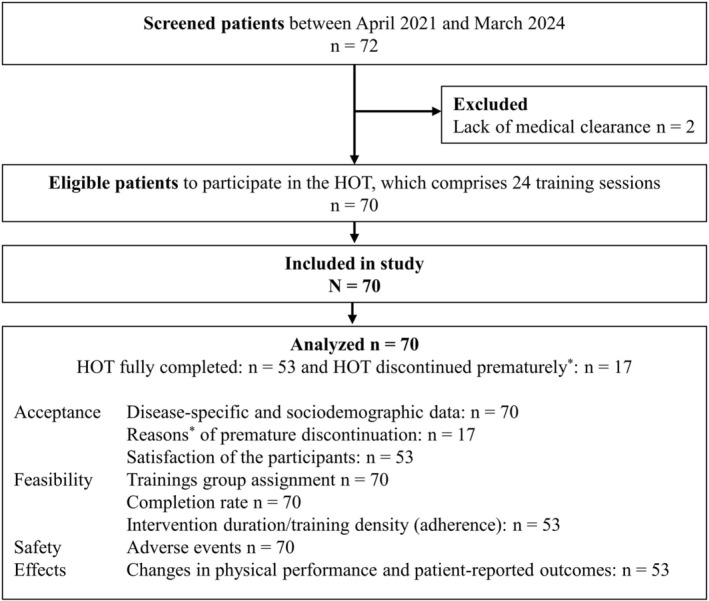
STROBE flow chart. HOT, Haematological and Oncological Training Therapy with Stationary Strength and Cardio Machines; *Reasons for discontinuation: health‐related reasons, for example, death, repeated hospitalisations, severe illness or therapy‐related symptoms that preclude intensive training, admission to inpatient rehabilitation, or personal reasons.

The STROBE flow chart for this study is shown in Figure 2.

Between April 2021 and March 2024, interest in participation exceeded capacity, and a waiting list was maintained throughout the entire period. At the time participation in HOT became possible, 70 of 72 patients received the medical clearance required and were included in the study. A total of 53 participants (76%) completed HOT, while 17 (24%) discontinued prematurely.

### 3.1 Participants' Sociodemographic and Clinical Data

The sociodemographic and clinical data of the participants are summarised in Table 3.

**TABLE 3:** Sociodemographic and clinical data.

Patients characteristics	*n*	Category	*n* (%) mean ± SD
Sex	70	Women Men	42 (60) 28 (40)
Age [years]	70		63 ± 12
Family status	70	Married/living with a partner Living alone	58 (83) 12 (17)
Professional status	68	Retired Working Unemployed	40 (59) 26 (38) 2 (3)
Sports activities before diagnosis	69	Continuously active In individual stages of life School sports only	35 (51) 30 (43) 4 (6)
Physical activity at inclusion (LSI)	69	Physically active Moderately active Insufficiently active	42 (61) 13 (19) 14 (20)
Body Mass Index [kg/m^2^]	68	< 18.5 18.5–24.9 25.0–29.9 ≥ 30.0	0 (0) 26 (38) 29 (43) 13 (19)
Place of treatment/aftercare	70	Practicing physician University Medical Center	43 (61) 27 (39)
Cancer type	70	Solid tumours Haematological neoplasms	43 (61) 27 (39)
Entity	70	Lymphomas incl. CLL and MM Gynecological and breast tumours Gastrointestinal tumours^b^ Myeloproliferative diseases Urogenital tumours Neuro‐oncological tumours Other	19 (27) 17 (24) 13 (19) 7 (10) 5 (7) 2 (3) 7 (10)
Therapy intention	70	Curative Palliative	36 (51) 34 (49)
Previous treatments^a^	70	Surgery Chemotherapy Radiotherapy Immunotherapy Endocrine therapy Radio‐iodine therapy	40 (57) 35 (50) 20 (29) 9 (13) 6 (9) 1 (1)
Therapy phase at inclusion	70	Undergoing treatment Follow‐up care^d^ Watch and wait Therapy break	40 (57) 25 (36) 4 (6) 1 (1)
Treatment during HOT	40	Chemotherapy Immunotherapy Endocrine therapy Chemo‐ and immunotherapy Targeted therapy Radio‐chemotherapy Radiotherapy Decision pending	20 (50) 6 (15) 6 (15) 3 (8) 2 (5) 1 (2) 1 (2) 1 (2)
Risk of CIPN		Yes	11 (16)
Side effects of oncologic therapy^a^	70	Fatigue CIPN Side effects of endocrine therapy Bone metastases Lymphedema Urinary incontinence Cachexia	40 (57) 25 (36) 7 (10) 7 (10) 6 (9) 4 (6) 2 (3)
Comorbidities^a^	70	Cardiovascular diseases Diabetes mellitus Osteoporosis Other^c^	29 (41) 10 (14) 5 (7) 17 (24)

Abbreviations: CIPN, chemotherapy‐induced peripheral neuropathy; CLL, chronic lymphocytic leukemia; HOT, Haematological and Oncological Training Therapy with Stationary Strength and Cardio Machines; LSI, Leisure Score Index; MM, multiple myeloma; *n*, number of patients; SD, standard deviation.

^a^ Multiple answers possible, according to the physicians' assessments.

^b^ Includes colorectal carcinomas, hepatobiliary tumours, esophagogastric tumours and pancreatic tumours.

^c^ Includes diseases without influence on the training program, for example, hay fever, hyperthyroidism, skin diseases.

^d^ The median time since diagnosis was 21 (14, 63) months. For 10 of the 25 patients in aftercare, the time since diagnosis had, in some cases, been well over 24 months.

Of the 70 participants included, 60% (*n* = 42) were female, and the mean age was 63 ± 12 years (range, 31–91 years). Prior to their cancer diagnosis, 94% (*n* = 65) of participants were already active in sports, 51% continuously and 43% during certain periods of their lives. At the time of enrolment, 61% (*n* = 42) were physically active, 19% (*n* = 13) were moderately active, and 20% (*n* = 14) were insufficiently active. Low‐intensity activities such as walking or yoga were most commonly reported.

The proportion of solid tumours versus haematological neoplasms was 61% (*n* = 43) versus 39% (*n* = 27). The most common diagnosis was lymphoma, including chronic lymphocytic leukaemia and multiple myeloma (27%, *n* = 19), followed by gynaecological/breast tumours (24%, *n* = 17) and gastrointestinal tumours (19%, *n* = 13). The median time since diagnosis was 21 (ranging between 7 and 51) months. At the time of participation, 57% (*n* = 40) of patients were undergoing active cancer treatment, with half of those (*n* = 20) receiving chemotherapy. Eleven participants were at risk of CIPN. According to physicians' assessments, 57% of participants (*n* = 40) exhibited symptoms of fatigue, 36% (*n* = 25) had CIPN, and 10% (*n* = 7) had bone metastases at the time of inclusion. The most common comorbidities were cardiovascular diseases (41%, *n* = 29) and diabetes mellitus (14%, *n* = 10).

No significant differences in sociodemographic or clinical characteristics were observed between completers and non‐completers, except that completers had higher baseline levels of neurotoxicity (*p* = 0.003) (Additional File 3).

### 3.2 Training Group Assignment

Based on clinical status and existing or expected side effects, 50% (*n* = 35) were assigned to the (adapted) BT group including SMT, 44% (*n* = 31) to the (adapted) BT group without SMT, and 6% (*n* = 4) received a training programme significantly different from BT, with or without SMT.

### 3.3 Satisfaction

All participants who completed HOT were satisfied with the programme and the support provided.

### 3.4 Safety: AEs

Over the 3‐year period, no SAEs occurred, and three AEs were recorded. In the first case, a patient experienced elbow pain after a 1‐RM chest press test. An X‐ray revealed calcification in the joint, and causality was assessed as “unlikely”. In the second case, a patient reported lower back pain following a 1‐RM back extension test; imaging revealed a pre‐existing osteoporotic vertebral fracture, and the event was considered “definitely related to the 1‐RM test”. In the third case, a patient developed aphasia after the first training session and was subsequently diagnosed with epilepsy; causality was assessed as “unlikely”. Accordingly, no AEs occurred during training (0 per 1000 patient sessions; 1364 patient sessions completed), whereas one AE occurred during performance diagnostics (8.1 per 1000 tests; 123 tests conducted).

### 3.5 Intervention Duration

From the 1st to the 24th training session, participants required a median of 108 (98, 130) days, with a range of 81 to 185 days. This corresponded to a median of 15 (14, 19) weeks and resulted in a median training density of 1.6 (1.3, 1.7) sessions per week.

The most common reasons for not adhering to the training schedule included receiving treatments (chemotherapy, radiation), severe side effects of medical therapy (e.g., nausea and dizziness), minor and major surgical procedures (e.g., port implantation), viral infections (e.g., COVID‐19), and holidays.

### 3.6 Premature Discontinuation: Reasons and Timing

During the observation period, 17 participants (24%) discontinued HOT prematurely; 12 (75%) did so for health‐related reasons, including symptom burden from medical therapy, repeated hospitalisations for relapse or new metastases, death, or short‐term admission to inpatient rehabilitation. Other reasons were mainly related to difficulties in accessing the training location, such as loss of driving ability or traffic disruptions due to road closures. Premature discontinuation occurred after a median of 5 (1, 8) training sessions.

### 3.7 Effects on Body Composition

The results of the bioelectrical impedance analysis, presented in Table 4, showed a significant increase in fat mass (ES = 0.49, *p* = 0.001) and body fat percentage (+1.4%p, ES = 0.55, *p* < 0.001) after completing HOT. Body mass index and skeletal muscle mass did not change significantly (*p* = 0.524 and *p* = 0.123, respectively). The median percentage change in muscle mass was −0.9% (−3.7%, 1.5%) (Additional File 4c). A slight increase in muscle mass was observed only in patients with CIPN (+0.4%), whereas the greatest decrease occurred in patients undergoing treatment (median −1.2%) (Additional File 5).

**TABLE 4:** Pre–post intervention changes in body composition and physical function.

Parameter	*n*	Pre	Post	Effect size	*p*
Body composition	49				
Body Mass Index [kg/m^2^]		26.3 (23.7, 29.2)	26.3 (23.5, 29.1)	0.09	0.524
Fat mass [kg]		26.1 ± 9.0	27.3 ± 9.5	0.49	0.001*
Body fat [%]		32.2 ± 8.4	33.6 ± 8.5	0.55	< 0.001**
Skeletal muscle mass [kg]		25.2 ± 5.7	24.9 ± 5.6	0.23	0.123
1‐ Repetition maximum [kg]					
Leg extension	45	61 ± 22	69 ± 18	0.41	0.011*
Leg curl	51	41 ± 17	48 ± 19	0.83	< 0.001**
Abdominal crunch	44	36 ± 8	44 ± 10	1.21	< 0.001**
Back extension	45	61 ± 21	69 ± 18	0.78	< 0.001**
Seated rowing	51	53 ± 17	62 ± 20	0.95	< 0.001**
Chest press	50	58 ± 20	67 ± 20	1.05	< 0.001**
Cycle ergometer test	49				
Max. power [W]		124 ± 39	135 ± 45	0.68	< 0.001**
Relative max. power [W/kg BW]		1.59 ± 0.48	1.72 ± 0.56	0.54	< 0.001**
RPE [6 to 20]		19 (17, 20)	19 (17, 19)	0.19	0.200
CR‐10‐scale [0 to 10]		2.0 (0.0, 3.0)	1.5 (0.0, 3.0)	0.10	0.485
Max. heart rate [bpm]		146 ± 27	148 ± 26	0.17	0.251

*Note:* Variables are presented as mean ± standard deviation or in case of non‐normality, as median (Q1, Q3). The paired *t*‐test or in case of non‐normality, Wilcoxon signed‐rank test was used to compare pre and post scores.

Abbreviations: bpm, beats per minute; BW, body weight; CR, Category‐Ratio; max., maximum; RPE, rate of perceived exertion.

* *p* ≤ 0.05.

** *p* < 0.001.

### 3.8 Effects on Physical Function

The numerical results of the pre–post comparison are presented in Table 4.

Post‐diagnostic assessments showed significant improvements in 1‐RM across all muscle groups (ES ≥ 0.41, *p* ≤ 0.011). Median percentage increases in 1‐RM ranged from 13% (leg extension) to 17% (abdominal crunch) (Additional File 4a). No significant correlations were found between changes in muscle mass and changes in 1‐RM (*p* > 0.05).

Subgroup analyses showed that increases in 1‐RM were comparable across all muscle groups in patients with solid tumours and haematological neoplasms, as well as in patients undergoing active cancer treatment and those in follow‐up care/watch‐and‐wait. Notably, the increase in strength in patients with CIPN was greater across all muscle groups than in patients without CIPN; however, with one exception (abdominal crunch, 29% vs. 14%, *p* = 0.040), these differences were not statistically significant. Regarding changes in 1‐RM, a significant sex difference in favour of men was observed only for the abdominal crunch (25% vs. 14%, *p* = 0.016) (Additional File 5).

Cardiorespiratory performance improved following HOT, with relative maximum power increasing from 1.59 ± 0.48 to 1.72 ± 0.56 W/kg body weight (ES = 0.54, *p* < 0.001), corresponding to a median relative increase of 7.5% (0.0%, 16.5%) (Additional File 4b). There were no significant differences between pre‐ and post‐tests in terms of RPE (*p* = 0.200), perceived leg pain (*p* = 0.485), or maximum heart rate (*p* = 0.251). Subgroup analyses showed that patients with CIPN had significantly greater improvements in relative maximum performance than those without CIPN (14.5% vs. 1.4%; *p* = 0.006). No significant differences were observed between patients with solid tumours and haematological neoplasms (8.5% vs. 1.4%, *p* = 0.678) or between women and men (4.0% vs. 11.0%, *p* = 0.387) (Additional File 5).

### 3.9 Effects on HRQoL, Symptoms, and Physical Activity

After completing HOT, participants showed significant improvements in global HRQoL (pre 50 (42, 67), post 67 (50, 75), ES = 0.56, *p* < 0.001), as well as on four of the five functional scales (*p* < 0.05), with ES ranging from small to medium (Table 5). The greatest improvements were observed in global HRQoL and physical function among patients with CIPN (median Δ of 16 and 13 points, respectively). Social functioning differed significantly between patients undergoing treatment and those in follow‐up care/watch‐and‐wait (median Δ of 17 vs. 0 points, *p* = 0.038) (Additional File 5).

**TABLE 5:** Pre–post intervention changes in health‐related quality of life, symptoms, and physical activity.

Parameter	Pre median (Q1, Q3)	Post median (Q1, Q3)	Effect size	*p*
QoL (EORTC QLQ‐C30^a^) *n* = 52				
Global QoL scale [0–100]	50 (42, 67)	67 (50, 75)	0.56	< 0.001**
Functional scales [0–100]				
Physical	73 (53, 84)	80 (60, 93)	0.50	< 0.001**
Role	50 (33, 67)	67 (50, 83)	0.53	< 0.001**
Emotional	58 (38, 83)	58 (50, 83)	0.31	0.026*
Cognitive	67 (50, 100)	75 (50, 100)	0.24	0.085
Social	50 (33, 75)	67 (50, 100)	0.51	< 0.001**
Symptom scales [0–100]				
Fatigue	56 (33, 67)	44 (33, 56)	0.39	0.005*
Nausea/vomiting	0 (0, 17)	0 (0, 13)	0.22	0.134
Pain	50 (17, 67)	33 (17, 50)	0.32	0.019*
Single items [0–100]				
Dyspnea	33 (0, 67)	33 (0, 67)	0.18	0.191
Insomnia	33 (33, 67)	33 (33, 67)	0.00	1.000
Appetite loss	0 (0, 33)	0 (0, 33)	0.11	0.453
Constipation	0 (0, 33)	0 (0, 33)	0.03	0.837
Diarrhea	0 (0, 0)	0 (0, 33)	0.20	0.148
Financial difficulties	0 (0, 33)	0 (0, 33)	0.01	0.959
Fatigue (MFI‐20^b^) *n* = 53				
Dimension of fatigue [4–20]				
General fatigue	13 (9, 17)	12 (8, 14)	0.43	0.001*
Physical fatigue	13 (11, 17)	10 (8, 14)	0.54	< 0.001**
Reduced activity	13 (8, 17)	10 (7, 14)	0.51	< 0.001**
Reduced motivation	9 (7, 11)	8 (6, 11)	0.27	0.054
Mental fatigue	8 (6, 14)	9 (6, 13)	0.09	0.541
Total fatigue score [20–100]	58 (44, 69)	48 (39, 62)	0.52	< 0.001**
Neuropathy (FACT/GOG‐Ntx‐13^c^) *n* = 21				
Neurotoxicity [0–52]	30 (24, 35)	34 (31, 39)	0.62	0.003*
Physical activity (GSLTPAQ^d^) *n* = 52				
Leisure Score Index [≥ 0]	30 (20, 51)	47 (26, 76)	0.46	< 0.001**

*Note:* The Wilcoxon signed‐rank test was used to compare pre and post scores.

Abbreviations: *n*, number of patients; QoL, quality of life.

^a^ Quality of Life questionnaire of cancer patients of the European Organization for Research and Treatment of Cancer, a high value on the scale “global QoL” and on the functional scales means a high degree of subjectively perceived health and a high assessment of the QoL or a high degree of performance and function. A high value in the symptom scales correlates with a high degree of complaints.

^b^ Multidimensional Fatigue Inventory, a high value correlates with a high degree of complaints.

^c^ Functional Assessment of Cancer Therapy/Gynecologic Oncology Group—Neurotoxicity 13 Item Version; a higher total score correlates with a lower neurotoxicity burden.

^d^ GSLTPAQ, Godin‐Shepard Leisure‐Time Physical Activity Questionnaire.

* *p* ≤ 0.05.

** *p* < 0.001.

According to the EORTC QLQ‐C30, fatigue improved significantly, with a mean decrease of 12 points (pre 56 (33, 67), post 44 (33, 56), ES = 0.39, *p* = 0.005). Similar improvements were observed in total fatigue using the MFI‐20, with a mean decrease of 10 points (pre 58 (44, 69), post 48 (39, 62), ES = 0.52, *p* < 0.001). These changes were mainly driven by reductions in “general fatigue”, “physical fatigue”, and “reduced activity”, which showed the highest baseline scores (median 13). No significant changes were observed in “reduced motivation” (*p* = 0.054) and “mental fatigue” (*p* = 0.541), which had lower baseline scores (median 9 and 8, respectively). A significantly greater reduction in fatigue was observed in patients with CIPN than in those without CIPN at baseline (median Δ −11 vs. −4 points, *p* = 0.020). No differences were observed between women and men (median Δ −6 points, *p* = 0.902) (Additional File 5).

The neurotoxicity score increased by 4 points from 30 (24, 35) to 34 (31, 39) (ES = 0.62, *p* = 0.003). Improvements were greater in men than in women, but the difference was not statistically significant (median Δ 7 vs. 3 points, *p* = 0.372) (Additional File 5).

Leisure‐time physical activity, assessed using the LSI, was significantly higher at the end of HOT than at the beginning (pre 30 (19, 51), post 47 (26, 76), ES = 0.46, *p* < 0.001).

## 4 Discussion

The aim of this observational study was to evaluate the acceptance, feasibility, and safety of HOT, a structured, individualised, cross‐entity training programme for patients with cancer, particularly those undergoing medical therapy and in the early phase of follow‐up care—and to assess its effects on physical performance and HRQoL. The three most important findings of this 3‐year real‐world data analysis were as follows: first, HOT is primarily used by patients with prior experience in sports; second, the selected eligibility and FITT criteria enable safe training regardless of cancer entity, treatment phase, side effects, or comorbidities; and third, patients with CIPN appear to benefit most from HOT.

### 4.1 Acceptance

Because of limited financial and staffing resources, HOT—a donation‐funded programme not covered by health insurance—had restricted capacity. Initiated in April 2021 under COVID‐19 hygiene regulations, it initially offered one training group (≤ 5 patients) twice weekly and expanded to two groups from summer 2022. Despite recruitment being limited to selected clinics and practices, demand consistently exceeded capacity.

The heterogeneity of participants in terms of sex, age, cancer entity, therapy phase, therapy intention, and treatments received before and during HOT reflects the reality of cancer care at the study site. A notable finding was the high proportion of participants with haematological neoplasms (39%) compared with solid tumours, which account for only 6%–8% of all cancers in general [32]. One possible explanation for this distribution is that the HOT programme was primarily advertised within UMR and affiliated practices, potentially introducing selection bias. By contrast, the proportion of gynaecological and breast tumours is markedly lower than that reported at the Cologne site, which recently published data from 8 years of integrating OTT into standard care (24% vs. ≥ 43%). This is likely because the women's clinic in Rostock is located at a different site.

Most participants had a prior history of exercise. At baseline, 80% were at least moderately physically active (LSI ≥ 14). Low‐intensity activities such as walking or yoga were reported most frequently. This high affinity for physical activity may explain the absence of a gap between eligible and included patients, the low dropout rate for personal reasons, and the higher training density compared with the Cologne OTT cohort (1.6 vs. 0.99 ± 0.5 sessions/week) [33]. The uniformly high satisfaction further supports the acceptability of the programme.

Nevertheless, motivating physically inactive patients to participate in exercise programmes remains challenging, despite evidence that post‐diagnosis physical activity is associated with reduced overall and cancer‐related mortality [10].

### 4.2 Feasibility

Except for four patients, all were able to complete the (adapted) BT with or without SMT without AEs, indicating that the eligibility criteria were appropriate. However, contraindications—particularly osteolysis and bone metastases—are carefully assessed during the initial consultation. Patients with extensive skeletal involvement were assigned individualised bodyweight exercise programmes.

The dropout rate was 24% over the first 3 years, exceeding rates reported in randomised controlled trials involving cancer survivors (~10%) [34] but substantially lower than in the Cologne OTT cohort (54%) [33]. Most discontinuations were due to disease progression or treatment‐related side effects, which are largely unpredictable. Baseline demographic and clinical characteristics did not allow identification of patients at higher risk of dropout.

Participants required a median of 15 weeks to complete HOT (24 sessions), corresponding to a capacity of approximately 30 patients per year. Increasing capacity and flexibility—for example, by expanding training days—may improve training density, reduce programme duration, and enhance effectiveness [33]. Compared with Cologne, where substantially greater resources enabled enrolment of up to 300 patients per year [33], our findings may be limited in generalisability. However, training density in our cohort was higher (1.6 vs. 0.99 sessions/week).

### 4.3 Safety

With a rate of 0 AEs per 1000 patient sessions, HOT enables safe training—in line with the literature [35, 36, 37]—when the defined eligibility and FITT criteria are followed. With an AE rate of 8.1 per 1000 performance diagnostics, these assessments represent the primary source of risk. The only HOT‐related AE occurred during a 1‐RM test in a patient with osteoporosis and a history of vertebral fracture. Therefore, osteoporosis should be considered a risk factor for fracture, and 1‐RM testing—particularly for the back extensors—should be avoided. Instead, resistance should be adjusted manually, and 1‐RM assessments should be omitted in these patients. Particular caution is also warranted in patients with bone metastases.

### 4.4 Effects of Intervention

The structured, individualised HOT programme was associated with improvement in strength, cardiorespiratory performance, fatigue, CIPN, and HRQoL, consistent with findings from randomised controlled trials [1, 2, 3, 4, 5, 6, 38, 39]. Given the moderate training frequency (1.6 sessions/week) and ongoing treatment in most participants, the observed moderate to high effect sizes are noteworthy. Possible reasons include: Structured exercise interventions in oncology studies that examine functional and physiological adaptations lasting between 6 and 24 weeks, with the majority of programs lasting 8 to 12 weeks [2, 40]. In contrast, the HOT programme was defined by a fixed number of 24 sessions rather than a fixed duration, resulting in a median intervention period of 15 weeks. This places the intervention at the upper end of commonly reported study durations and may have allowed for more gradual physiological and functional adaptations, as well as additional changes in habitual physical activity. However, participants were instructed to maintain habitual activity—and LSI increased (median 30 vs. 47)—the effects cannot be attributed solely to the intervention. In addition, natural recovery, changes in systemic therapy or supportive care could also contribute to the observed changes. This is particularly relevant for patients who have completed chemotherapy or undergone major surgery, as longitudinal studies indicate partial recovery of physical function and symptom burden during the post‐treatment phase, although impairments may persist compared with pre‐diagnosis or healthy populations [41]. This also applies to the present cohort, in which 57% of patients had undergone surgery and 50% had received chemotherapy prior to enrolment in the HOT programme. However, recovery trajectories are highly variable and often incomplete, suggesting that spontaneous recovery alone is unlikely to account for the magnitude of the observed effects [41, 42, 43]. It should also be noted, that only completers were analysed, introducing potential attrition bias and likely overestimating effects [44, 45].

Strength gains (13% to 17%) likely reflect adherence to training principles such as specificity and progression [46], as well as evidence‐based set/repetition schemes [13]. Although no established minimal important difference (MID) exists for muscle strength, and higher thresholds (~27%) have been proposed [47], smaller improvements may still be clinically meaningful when maintaining physical function is a primary goal. Minimal physiological adaptations and the lack of association between muscle mass and strength gains suggest predominantly neural adaptations and motor learning effects [48, 49]. The fact that physiological/anabolic adaptations were limited despite targeted hypertrophy training is consistent with known treatment‐induced impairments in muscle metabolism and neuromuscular function [50, 51, 52, 53]. The pronounced improvements in patients with CIPN further support this, indicating enhanced neuromuscular coordination and control, potentially supported by SMT [6, 38]. In addition, exercise‐induced neurobiological adaptations—such as reduced neuroinflammation, modulation of neurotransmitter systems, and enhanced endogenous pain inhibition—may have contributed to symptom relief in this subgroup [54].

The observed increase in fat mass across all subgroups was somewhat unexpected. Given the relatively high activity levels of the participants, reduced physical activity alone is unlikely to explain this finding. Instead, treatment‐related metabolic alterations—including changes in energy metabolism, systemic inflammation, and hormonal regulation—may contribute to increased fat accumulation, as described in cancer patients undergoing chemotherapy and other treatments [55, 56]. In line with this, a large population‐based study demonstrated that the prevalence of obesity increases more rapidly among cancer survivors compared to individuals without a history of cancer [57]. Considering that 62% of our participants were already overweight or obese at baseline, and given the well‐established association between obesity, adverse treatment outcomes, and comorbidities, greater emphasis should be placed on weight management and control in this patient population.

Cardiorespiratory improvements likely reflect both HOT's aerobic intervals and activities outside the programme. The observed improvements may be explained by exercise‐induced adaptations across multiple components of the oxygen transport system, which are known to be impaired by cancer and its treatment [58]. VO_2_max was not assessed, but the Borg scale proved a practical tool for intensity control in routine care. Despite the absence of a defined MID, improvements in aerobic capacity are known to be associated with clinically relevant outcomes, including reduced mortality risk [59].

Fatigue reduction of 12 or 10 points (EORTC QLQ‐C30 or MFI‐20) appears to be clinically relevant [60, 61] and aligns with established FITT recommendations: ≥ 12 weeks, twice‐weekly combined aerobic and resistance training at moderate to high intensity [13]. It was evident that participants' motivation was not significantly reduced despite general physical tiredness, which can be a barrier to activity [62]. This may explain the predominance of previously active participants. Motivation is a key factor for sustained participation in physical training [63, 64, 65]. SMT showed expected benefits for CIPN [5], though its isolated effects remain unclear [38]. SMT aims to improve proprioception, neuromuscular control, and balance, thereby compensating for sensory deficits, reducing fall risk, and enhancing functional performance in daily activities [6, 30, 66]. However, as outcomes were assessed only via PROs, which primarily reflect symptoms such as pain and paraesthesia, no direct conclusions can be drawn regarding changes in proprioception or balance. Notably, the observed improvements in maximal strength—particularly in patients with CIPN—may indicate enhanced neuromuscular activation, although this remains speculative. Given the pragmatic, real‐world design, the focus was on feasibility and implementation within routine care rather than on disentangling mechanism‐specific effects. Accordingly, and in line with current evidence [6, 38, 67], SMT should be considered for patients at risk.

Improved HRQoL likely reflects the physical health gains achieved, in line with meta‐analytic findings [68]. Considering that MID values for the EORTC QLQ‐C30 typically range between 5 and 10 points across scales and cancer entities [61], the present results suggest that the observed improvements in global HRQoL, as well as in physical, role, and social functioning, are clinically meaningful. Notably, in patients with CIPN, the objective gains in muscular strength and cardiorespiratory fitness, accompanied by symptom relief, are also reflected in PROs. Overall, the greatest improvements in physical performance and symptom burden were observed in patients with pre‐existing CIPN who received SMT in addition to BT (combined strength and endurance training). Previous studies suggest that both SMT alone and multimodal training programs consisting of SMT, strength training, and aerobic training improve CIPN‐related symptoms and function. These improvements appear to be primarily driven by compensatory neuromuscular mechanisms/adaptations rather than structural nerve regeneration [6, 66, 69]. However, the underlying mechanisms have not yet been fully elucidated. Future studies should therefore investigate whether supplementing BT with SMT can also improve training responses in patients without CIPN.

### 4.5 Limitations

Limitations of this study include the single‐arm design without a control group and the relatively small sample size, particularly in subgroup analyses. Only participants who completed the intervention were included in the analyses. Patients whose health status deteriorated during the intervention and who discontinued prematurely were not considered, introducing potential attrition bias and possibly leading to an overestimation of the observed effects [44, 45]. Furthermore, the study population consisted predominantly of physically active individuals, indicating potential selection bias. This may have contributed to the high adherence observed and limits the generalisability of the findings, particularly to less active patient populations. Finally, it should be noted that a wide range of endpoints was examined. Given the large number of statistical tests performed, the risk of type I error cannot be excluded. As no formal correction for multiple testing was applied, the results should be interpreted with caution. In this context, the analyses should be considered exploratory and hypothesis‐generating rather than confirmatory. Further studies are required to confirm and extend these preliminary findings.

A potential limitation is the use of 1‐RM–based strength assessment and prescription. Although widely used in exercise oncology, fixed percentages of 1‐RM may not reflect comparable relative intensities across individuals, potentially introducing variability in training load and strength outcomes [70]. A further limitation relates to the assessment of cardiorespiratory fitness. This was evaluated using a maximal cycle ergometer test with (relative) peak power output as the outcome. Although peak power output has been shown to correlate with aerobic capacity and is commonly used as a pragmatic alternative in clinical and exercise oncology settings, it does not provide a direct measure of VO_2_peak, which is considered the gold standard [71].

As a supportive therapy, HOT primarily aims to improve patients' HRQoL, a multidimensional construct. However, not all relevant dimensions were equally captured in the present assessment. In particular, psychological aspects such as anxiety, depression, and distress, as well as social interactions during small‐group training, were insufficiently assessed. Future studies should consider incorporating additional validated instruments, such as the Hospital Anxiety and Depression Scale, the Distress Thermometer, or a group questionnaire.

## 5 Conclusion

The HOT programme, based on the OTT concept, was well accepted by patients of both sexes and across a wide range of cancer entities. The findings demonstrate that a supervised, moderate‐ to vigorous‐intensity combined resistance and endurance training programme can be safely implemented before, during, and after cancer treatment when appropriate screening procedures are applied. Particular caution is warranted for 1‐RM testing in patients with osteoporosis or bone metastases, where individualised adjustments are recommended.

With a training adherence of at least 1.6 sessions per week, clinically meaningful improvements in physical function and HRQoL can be expected. The results further highlight the value of multimodal exercise interventions, particularly for patients with CIPN. While most guidelines are based on solid tumour data [13], our findings suggest that such approaches are also feasible and beneficial in patients with haematological neoplasms.

However, the current voluntary participation model predominantly attracts physically active individuals, limiting generalisability. In addition, restricted financial and staffing resources constrain programme capacity and fail to meet demand. Notably, the implementation, utilisation, adherence, and resulting effects of such exercise programmes appear to be influenced by regional factors, with differences observed between metropolitan areas (e.g., Cologne) and more rural regions (e.g., Rostock/M‐V). Future efforts should therefore focus on expanding access, addressing regional disparities, and developing targeted strategies to engage less active patients, thereby ensuring equitable participation in exercise oncology programmes.

## Author Contributions

**Sabine Felser:** conceptualization (lead), data curation (lead), formal analysis (lead), funding acquisition (lead), investigation (lead), project administration (lead), supervision (lead), writing – original draft (lead). **Maya Engel:** formal analysis (equal), writing – original draft (equal). **Christina Grosse‐Thie:** conceptualization (equal), investigation (equal), project administration (equal), writing – review and editing (equal). **Brigitte Kragl:** conceptualization (equal), writing – review and editing (equal). **Larissa Henze:** investigation (equal), writing – review and editing (equal). **Imke Albrecht:** investigation (supporting), writing – review and editing (equal). **Hans Lampe:** investigation (equal), writing – review and editing (equal). **Susanne Fischer:** data curation (equal), software (lead), writing – review and editing (equal). **Ulrich Langenkamp:** investigation (equal), writing – review and editing (equal). **Christian Junghanss:** conceptualization (equal), funding acquisition (equal), supervision (equal), writing – review and editing (equal).

## Funding

This work was supported by the Cancer Society of M‐V under grant number “Project 13”. The funder had no role in the study design, data collection and analysis, decision to publish, or preparation of the manuscript.

## Ethics Statement

The study was conducted in accordance with the Declaration of Helsinki [22] and approved by the Ethics Committee of the University of Rostock (A2020‐0211). The study was registered in the German Clinical Trials Register (DRKS00023912), and all participants provided written informed consent.

## Consent

The authors have nothing to report.

## Conflicts of Interest

The authors declare no conflicts of interest.

## Supporting information


**Supplement 1:** Live feedback on movement execution.

**Supplement 2:** Procedures for Haematological and Oncological Training Therapy using stationary strength and cardio machines (HOT).

**Supplement 3:** Characteristics of completers versus non‐completers.

**Supplement 4:** Percentage change in (a) 1‐repetition maximum in the six strengthening exercises of the basic training, (b) relative maximum power on the cycle ergometer, and (c) skeletal muscle mass after completion Hematological and Oncological Training Therapy using stationary strength and cardio machines.

**Supplement 5:** Changes pre‐post intervention.

## Data Availability

The raw data supporting the conclusions of this article will be made available by the authors without undue reservation. The generated and analysed datasets are available in the NFDI4Health repository: https://ldh.mediz‐rostock.imise.uni‐leipzig.de/projects/19.

## References

[1] M. G. Sweegers , T. M. Altenburg , M. J. Chinapaw , et al., “Which Exercise Prescriptions Improve Quality of Life and Physical Function in Patients With Cancer During and Following Treatment? A Systematic Review and Meta‐Analysis of Randomised Controlled Trials,” British Journal of Sports Medicine 52, no. 8 (2018): 505–513. 28954800 10.1136/bjsports-2017-097891

[2] Q. Zhang , Y. Gao , W. Wang , X. Zhao , J. Yu , and H. Huang , “Effect of Resistance Exercise on Physical Fitness, Quality of Life, and Fatigue in Patients With Cancer: A Systematic Review,” Frontiers in Oncology 14 (2024): 1393902. 39099690 10.3389/fonc.2024.1393902 PMC11294253

[3] B. J. Baguley , K. A. Bolam , O. R. L. Wright , and T. L. Skinner , “The Effect of Nutrition Therapy and Exercise on Cancer‐Related Fatigue and Quality of Life in Men With Prostate Cancer: A Systematic Review,” Nutrients 9, no. 9 (2017): 1003. 28895922 10.3390/nu9091003 PMC5622763

[4] H.‐J. Zhou , T. Wang , Y.‐Z. Xu , et al., “Effects of Exercise Interventions on Cancer‐Related Fatigue in Breast Cancer Patients: An Overview of Systematic Reviews,” Supportive Care in Cancer 30, no. 12 (2022): 10421–10440. 36326908 10.1007/s00520-022-07389-5 PMC9715478

[5] Y. Huang , T. Tan , L. Liu , et al., “Exercise for Reducing Chemotherapy‐Induced Peripheral Neuropathy: A Systematic Review and Meta‐Analysis of Randomized Controlled Trials,” Frontiers in Neurology 14 (2023): 1252259. 38283674 10.3389/fneur.2023.1252259 PMC10813204

[6] F. Streckmann , T. Elter , H. C. Lehmann , et al., “Preventive Effect of Neuromuscular Training on Chemotherapy‐Induced Neuropathy: A Randomized Clinical Trial,” JAMA Internal Medicine 184, no. 9 (2024): 1046–1053. 38949824 10.1001/jamainternmed.2024.2354 PMC11217888

[7] V. Martínez‐Vizcaíno , I. Cavero‐Redondo , S. Reina‐Gutiérrez , et al., “Comparative Effects of Different Types of Exercise on Health‐Related Quality of Life During and After Active Cancer Treatment: A Systematic Review and Network Meta‐Analysis,” Journal of Sport and Health Science 12, no. 6 (2023): 726–738. 36736726 10.1016/j.jshs.2023.01.002 PMC10658325

[8] L. Yang , A. R. Morielli , E. Heer , et al., “Effects of Exercise on Cancer Treatment Efficacy: A Systematic Review of Preclinical and Clinical Studies,” Cancer Research 81, no. 19 (2021): 4889–4895. 34215623 10.1158/0008-5472.CAN-21-1258 PMC9397632

[9] P. Cormie , E. M. Zopf , X. Zhang , and K. H. Schmitz , “The Impact of Exercise on Cancer Mortality, Recurrence, and Treatment‐Related Adverse Effects,” Epidemiologic Reviews 39, no. 1 (2017): 71–92. 28453622 10.1093/epirev/mxx007

[10] A. V. Patel , C. M. Friedenreich , S. C. Moore , et al., “American College of Sports Medicine Roundtable Report on Physical Activity, Sedentary Behavior, and Cancer Prevention and Control,” Medicine and Science in Sports and Exercise 51, no. 11 (2019): 2391–2402. 31626056 10.1249/MSS.0000000000002117 PMC6814265

[11] P. Hojman , J. Gehl , J. F. Christensen , and B. K. Pedersen , “Molecular Mechanisms Linking Exercise to Cancer Prevention and Treatment,” Cell Metabolism 27, no. 1 (2018): 10–21. 29056514 10.1016/j.cmet.2017.09.015

[12] R. Thomas , S. A. Kenfield , Y. Yanagisawa , and R. U. Newton , “Why Exercise Has a Crucial Role in Cancer Prevention, Risk Reduction and Improved Outcomes,” British Medical Bulletin 139, no. 1 (2021): 100–119. 34426823 10.1093/bmb/ldab019 PMC8431973

[13] K. L. Campbell , K. M. Winters‐Stone , J. Wiskemann , et al., “Exercise Guidelines for Cancer Survivors: Consensus Statement From International Multidisciplinary Roundtable,” Medicine and Science in Sports and Exercise 51, no. 11 (2019): 2375–2390. 31626055 10.1249/MSS.0000000000002116 PMC8576825

[14] P. Cormie , M. Atkinson , L. Bucci , et al., “Clinical Oncology Society of Australia Position Statement on Exercise in Cancer Care,” Medical Journal of Australia 209, no. 4 (2018): 184–187. 29719196 10.5694/mja18.00199

[15] K. H. Schmitz , A. M. Campbell , M. M. Stuiver , et al., “Exercise Is Medicine in Oncology: Engaging Clinicians to Help Patients Move Through Cancer,” CA: A Cancer Journal for Clinicians 69, no. 6 (2019): 468–484. 31617590 10.3322/caac.21579 PMC7896280

[16] Bundesarbeitsgemeinschaft für Rehabilitation e. V , “Rehabilitationssport und Funktionstraining: Rahmenvereinbarung,” accessed April 1, 2026, https://www.bar‐frankfurt.de/fileadmin/dateiliste/_publikationen/reha_vereinbarungen/vereinbarung/downloads/rahmenvereinbarung_ueber_den_rehabilitationssport_und_das_funktionstraining.pdf.

[17] F. T. Baumann , M. Hallek , J. Meyer , D. A. Galvão , W. Bloch , and T. Elter , “Onkologische Trainings‐ und Bewegungstherapie (OTT). [Evidence and Recommendations for Oncologic Clinical Exercise—A Personalized Treatment Concept for Cancer Patients] [ger],” Deutsche medizinische Wochenschrift (1946) 140, no. 19 (2015): 1457–1461. 26402184 10.1055/s-0041-104465

[18] M. S. Fragala , E. L. Cadore , S. Dorgo , et al., “Resistance Training for Older Adults: Position Statement From the National Strength and Conditioning Association,” Journal of Strength and Conditioning Research 33, no. 8 (2019): 2019–2052. 31343601 10.1519/JSC.0000000000003230

[19] G. G. Haff , “Roundtable Discussion: Machines Versus Free Weights,” Strength and Conditioning Journal 22, no. 6 (2000): 18–30.

[20] M. E. Haugen , F. T. Vårvik , S. Larsen , A. S. Haugen , R. van den Tillaar , and T. Bjørnsen , “Effect of Free‐Weight vs. Machine‐Based Strength Training on Maximal Strength, Hypertrophy and Jump Performance – A Systematic Review and Meta‐Analysis,” BMC Sports Science, Medicine & Rehabilitation 15, no. 1 (2023): 1–20. 10.1186/s13102-023-00713-4 PMC10426227 37582807

[21] S. Bundesamt , Bevölkerungsdichte in Deutschland nach Bundesländern zum 31 (2024), accessed Mar 31, 2026, https://de.statista.com/statistik/daten/studie/1242/umfrage/bevoelkerungsdichte‐in‐deutschland‐nach‐bundeslaendern/.

[22] World Medical Association , “Declaration of Helsinki: Ethical Principles for Medical Research Involving Human Subjects,” Journal of the American Medical Association 310, no. 20 (2013): 2191–2194. 24141714 10.1001/jama.2013.281053

[23] M. M. Wefelnberg , T. Niels , U. Holtick , F. Jundt , C. Scheid , and F. T. Baumann , “Clinical Exercise Therapy Program With Multiple Myeloma Patients: Impacts on Feasibility, Adherence and Efficacy,” Supportive Care in Cancer 30, no. 11 (2022): 9615–9623. 36190557 10.1007/s00520-022-07369-9 PMC9633464

[24] G. A. Borg , “Psychophysical Bases of Perceived Exertion,” Medicine and Science in Sports and Exercise 14, no. 5 (1982): 377–381. 7154893

[25] H. Löllgen and D. Leyk , “Exercise Testing in Sports Medicine,” Deutsches Arzteblatt International 115, no. 24 (2018): 409–416. 29968559 10.3238/arztebl.2018.0409 PMC6050434

[26] E. Borg , G. Borg , K. Larsson , M. Letzter , and B.‐M. Sundblad , “An Index for Breathlessness and Leg Fatigue,” Scandinavian Journal of Medicine & Science in Sports 20, no. 4 (2010): 644–650. 19602182 10.1111/j.1600-0838.2009.00985.x

[27] N. K. Aaronson , S. Ahmedzai , B. Bergman , et al., “The European Organization for Research and Treatment of Cancer QLQ‐C30: A Quality‐Of‐Life Instrument for Use in International Clinical Trials in Oncology,” Journal of the National Cancer Institute 85, no. 5 (1993): 365–376. 8433390 10.1093/jnci/85.5.365

[28] K. Cocks , J. R. Wells , C. Johnson , et al., “Content Validity of the EORTC Quality of Life Questionnaire QLQ‐C30 for Use in Cancer,” European Journal of Cancer (Oxford, England: 1990) 178 (2023): 128–138. 36436330 10.1016/j.ejca.2022.10.026

[29] E. M. Smets , B. Garssen , B. Bonke , and H. J. C. de , “The Multidimensional Fatigue Inventory (MFI) Psychometric Qualities of an Instrument to Assess Fatigue,” Journal of Psychosomatic Research 39, no. 3 (1995): 315–325. 7636775 10.1016/0022-3999(94)00125-o

[30] E. A. Calhoun , E. E. Welshman , C.‐H. Chang , et al., “Psychometric Evaluation of the Functional Assessment of Cancer Therapy/Gynecologic Oncology Group‐Neurotoxicity (Fact/GOG‐Ntx) Questionnaire for Patients Receiving Systemic Chemotherapy,” International Journal of Gynecological Cancer: Official Journal of the International Gynecological Cancer Society 13, no. 6 (2003): 741–748. 14675309 10.1111/j.1525-1438.2003.13603.x

[31] S. Amireault and G. Godin , “The Godin‐Shephard Leisure‐Time Physical Activity Questionnaire: Validity Evidence Supporting Its Use for Classifying Healthy Adults Into Active and Insufficiently Active Categories,” Perceptual and Motor Skills 120, no. 2 (2015): 604–622. 25799030 10.2466/03.27.PMS.120v19x7

[32] Cécile Ronckers , Claudia Spix , Claudia Trübenbach , et al., Robert Koch‐Institut und die Gesellschaft der epidemiologischen Krebsregister in Deutschland e.V. RKI—Krebs in Deutschland für 2019/2020, 14th ed. (Robert Koch‐Institut, 2023).

[33] T. Sonntag , A. Safi , V. Coutellier , et al., “Innovative Exercise in Routine Cancer Care: Insights From Eight Years of Integrated Oncological Exercise Therapy (OTT),” Sports Medicine—Open 12, no. 1 (2026): 22. 41779285 10.1186/s40798-026-00988-0 PMC12961029

[34] B. Western , A. Ivarsson , I. Vistad , et al., “Dropout From Exercise Trials Among Cancer Survivors‐An Individual Patient Data Meta‐Analysis From the POLARIS Study,” Scandinavian Journal of Medicine & Science in Sports 34, no. 2 (2024): e14575. 38339809 10.1111/sms.14575

[35] D. Clauss , F. Quirmbach , J. Wiskemann , and F. Rosenberger , “Adverse Events beim Training mit onkologischen Patienten: Wie sicher ist das Training außerhalb klinischer Studien?,” B & G 35, no. 4 (2019): 194–201.

[36] R. Heywood , A. L. McCarthy , and T. L. Skinner , “Safety and Feasibility of Exercise Interventions in Patients With Advanced Cancer: A Systematic Review,” Supportive Care in Cancer: Official Journal of the Multinational Association of Supportive Care in Cancer 25, no. 10 (2017): 3031–3050. 28741176 10.1007/s00520-017-3827-0

[37] B. Singh , R. R. Spence , M. L. Steele , C. X. Sandler , J. M. Peake , and S. C. Hayes , “A Systematic Review and Meta‐Analysis of the Safety, Feasibility, and Effect of Exercise in Women With Stage II+ Breast Cancer,” Archives of Physical Medicine and Rehabilitation 99, no. 12 (2018): 2621–2636. 29730319 10.1016/j.apmr.2018.03.026

[38] N. Nakagawa , S. Yamamoto , A. Hanai , A. Oiwa , and H. Arao , “Exercise Intervention for the Management of Chemotherapy‐Induced Peripheral Neuropathy: A Systematic Review and Network Meta‐Analysis,” Frontiers in Neurology 15 (2024): 1346099. 38352137 10.3389/fneur.2024.1346099 PMC10861771

[39] H. van Waart , M. M. Stuiver , W. H. van Harten , et al., “Effect of Low‐Intensity Physical Activity and Moderate‐ to High‐Intensity Physical Exercise During Adjuvant Chemotherapy on Physical Fitness, Fatigue, and Chemotherapy Completion Rates: Results of the PACES Randomized Clinical Trial,” Journal of Clinical Oncology: Official Journal of the American Society of Clinical Oncology 33, no. 17 (2015): 1918–1927. 25918291 10.1200/JCO.2014.59.1081

[40] L. N. de , T. Niels , M. Tewes , and M. Götte , “A Systematic Review of the Safety, Feasibility and Benefits of Exercise for Patients With Advanced Cancer,” Cancers 13, no. 17 (2021): 4478. 34503288 10.3390/cancers13174478 PMC8430671

[41] E. M. Cespedes Feliciano , S. Vasan , J. Luo , et al., “Long‐Term Trajectories of Physical Function Decline in Women With and Without Cancer,” JAMA Oncology 9, no. 3 (2023): 395–403. 36656572 10.1001/jamaoncol.2022.6881 PMC9857739

[42] I. Vaz‐Luis , A. Di Meglio , J. Havas , et al., “Long‐Term Longitudinal Patterns of Patient‐Reported Fatigue After Breast Cancer: A Group‐Based Trajectory Analysis,” Journal of Clinical Oncology: Official Journal of the American Society of Clinical Oncology 40, no. 19 (2022): 2148–2162. 35290073 10.1200/JCO.21.01958 PMC9242405

[43] C. Bodelon , M. Masters , D. E. Bloodworth , et al., “Physical Health Decline After Chemotherapy or Endocrine Therapy in Breast Cancer Survivors,” JAMA Network Open 8, no. 2 (2025): e2462365. 40019757 10.1001/jamanetworkopen.2024.62365 PMC11871543

[44] A. Lewin , R. Brondeel , T. Benmarhnia , F. Thomas , and B. Chaix , “Attrition Bias Related to Missing Outcome Data: A Longitudinal Simulation Study,” Epidemiology (Cambridge, Mass.) 29, no. 1 (2018): 87–95. 28926372 10.1097/EDE.0000000000000755

[45] K. Gustavson , “Attrition and Generalizability in Longitudinal Studies: Findings From a 15‐Year Population‐Based Study and a Monte Carlo Simulation Study,” BMC Public Health 12, no. 1 (2012): 1–11. 23107281 10.1186/1471-2458-12-918 PMC3503744

[46] C. M. Fairman , P. N. Hyde , and B. C. Focht , “Resistance Training Interventions Across the Cancer Control Continuum: A Systematic Review of the Implementation of Resistance Training Principles,” British Journal of Sports Medicine 51, no. 8 (2017): 677–685. 27986761 10.1136/bjsports-2016-096537

[47] A. Oliveira , P. Rebelo , C. Paixão , et al., “Minimal Clinically Important Difference for Quadriceps Muscle Strength in People With COPD Following Pulmonary Rehabilitation,” COPD: Journal of Chronic Obstructive Pulmonary Disease 18, no. 1 (2021): 35–44. 33533285 10.1080/15412555.2021.1874897

[48] R. M. Enoka , Neuromechanics of Human Movement (Human Kinetics Library, 2015).

[49] D. A. Gabriel , G. Kamen , and G. Frost , “Neural Adaptations to Resistive Exercise: Mechanisms and Recommendations for Training Practices,” Sports Medicine (Auckland, N.Z.) 36, no. 2 (2006): 133–149. 16464122 10.2165/00007256-200636020-00004

[50] J. S. Damrauer , M. E. Stadler , S. Acharyya , A. S. Baldwin , M. E. Couch , and D. C. Guttridge , “Chemotherapy‐Induced Muscle Wasting: Association With NF‐κB and Cancer Cachexia,” European Journal of Translational Myology 28, no. 2 (2018): 7590. 29991992 10.4081/ejtm.2018.7590 PMC6036305

[51] J. M. Garcia , J. P. Cata , P. M. Dougherty , and R. G. Smith , “Ghrelin Prevents Cisplatin‐Induced Mechanical Hyperalgesia and Cachexia,” Endocrinology 149, no. 2 (2008): 455–460. 17962345 10.1210/en.2007-0828 PMC2219295

[52] T. Le Bricon , S. Gugins , L. Cynober , and V. E. Baracos , “Negative Impact of Cancer Chemotherapy on Protein Metabolism in Healthy and Tumor‐Bearing Rats,” Metabolism: Clinical and Experimental 44, no. 10 (1995): 1340–1348. 7476295 10.1016/0026-0495(95)90040-3

[53] A. Martin , Y. S. Gallot , and D. Freyssenet , “Molecular Mechanisms of Cancer Cachexia‐Related Loss of Skeletal Muscle Mass: Data Analysis From Preclinical and Clinical Studies,” Journal of Cachexia, Sarcopenia and Muscle 14, no. 3 (2023): 1150–1167. 36864755 10.1002/jcsm.13073 PMC10235899

[54] J. Loureiro , J. T. Costa‐Pereira , D. H. Pozza , and I. Tavares , “The Power of Movement: How Exercise Influences Chemotherapy‐Induced Peripheral Neuropathy,” Biomedicine 13, no. 5 (2025): 1103. 10.3390/biomedicines13051103 PMC12109246 40426930

[55] P. T. Bradshaw , “Body Composition and Cancer Survival: A Narrative Review,” British Journal of Cancer 130, no. 2 (2024): 176–183. 37891197 10.1038/s41416-023-02470-0 PMC10803330

[56] W. Demark‐Wahnefried , B. L. Peterson , E. P. Winer , et al., “Changes in Weight, Body Composition, and Factors Influencing Energy Balance Among Premenopausal Breast Cancer Patients Receiving Adjuvant Chemotherapy,” Journal of Clinical Oncology: Official Journal of the American Society of Clinical Oncology 19, no. 9 (2001): 2381–2389. 11331316 10.1200/JCO.2001.19.9.2381

[57] H. Greenlee , Z. Shi , C. L. Sardo Molmenti , A. Rundle , and W. Y. Tsai , “Trends in Obesity Prevalence in Adults With a History of Cancer: Results From the US National Health Interview Survey, 1997 to 2014,” Journal of Clinical Oncology: Official Journal of the American Society of Clinical Oncology 34, no. 26 (2016): 3133–3140. 27458295 10.1200/JCO.2016.66.4391 PMC5012707

[58] L. W. Jones , N. D. Eves , M. Haykowsky , S. J. Freedland , and J. R. Mackey , “Exercise Intolerance in Cancer and the Role of Exercise Therapy to Reverse Dysfunction,” Lancet. Oncology 10, no. 6 (2009): 598–605. 19482248 10.1016/S1470-2045(09)70031-2

[59] F. Bettariga , D. A. Galvao , D. R. Taaffe , et al., “Association of Muscle Strength and Cardiorespiratory Fitness With All‐Cause and Cancer‐Specific Mortality in Patients Diagnosed With Cancer: A Systematic Review With Meta‐Analysis,” British Journal of Sports Medicine 59, no. 10 (2025): 722–732. 39837589 10.1136/bjsports-2024-108671

[60] J. Z. Musoro , C. Coens , F. Fiteni , et al., “Minimally Important Differences for Interpreting EORTC QLQ‐C30 Scores in Patients With Advanced Breast Cancer,” JNCI Cancer Spectrum 3, no. 3 (2019): pkz037. 32328553 10.1093/jncics/pkz037 PMC7050000

[61] J. Z. Musoro , C. Coens , M. A. G. Sprangers , et al., “Minimally Important Differences for Interpreting EORTC QLQ‐C30 Change Scores Over Time: A Synthesis Across 21 Clinical Trials Involving Nine Different Cancer Types,” European Journal of Cancer (Oxford, England: 1990) 188 (2023): 171–182. 37257278 10.1016/j.ejca.2023.04.027

[62] S. A. D. Romero , J. C. Brown , J. M. Bauml , et al., “Barriers to Physical Activity: A Study of Academic and Community Cancer Survivors With Pain,” Journal of Cancer Survivorship: Research and Practice 12, no. 6 (2018): 744–752. 30182150 10.1007/s11764-018-0711-y PMC6461363

[63] B. A. Bushman , ed., ACSM's Complete Guide to Fitness & Health (Champaign IL, 2017).

[64] B. A. Bushman , “Promoting Healthy Habits: Getting Started and Staying Motivated,” in ACSM's Complete Guide to Fitness & Health, ed. B. A. Bushman (Human Kinetics, 2017), 61–76.

[65] S. Felser , M. Behrens , H. Lampe , et al., “Motivation and Preferences of Cancer Patients to Perform Physical Training,” European Journal of Cancer Care 29, no. 4 (2020): e13246. 32476203 10.1111/ecc.13246

[66] F. Streckmann , M. Balke , G. Cavaletti , et al., “Exercise and Neuropathy,” Systematic Review With Meta‐Analysis. Sports Medicine 52 (2021): 1–23. 10.1007/s40279-021-01596-6 34964950

[67] R. Brownson‐Smith , S. T. Orange , N. Cresti , K. Hunt , J. Saxton , and J. Temesi , “Effect of Exercise Before and/or During Taxane‐Containing Chemotherapy Treatment on Chemotherapy‐Induced Peripheral Neuropathy Symptoms in Women With Breast Cancer: Systematic Review and Meta‐Analysis,” Journal of Cancer Survivorship: Research and Practice 19 (2023): 78–96. 37615928 10.1007/s11764-023-01450-w PMC11813970

[68] J. K. W. Gerritsen and A. J. P. E. Vincent , “Exercise Improves Quality of Life in Patients With Cancer: A Systematic Review and Meta‐Analysis of Randomised Controlled Trials,” British Journal of Sports Medicine 50, no. 13 (2016): 796–803. 26719503 10.1136/bjsports-2015-094787

[69] A. Cao , B. Cartmel , F.‐Y. Li , et al., “Effect of Exercise on Chemotherapy‐Induced Peripheral Neuropathy Among Patients Treated for Ovarian Cancer: A Secondary Analysis of a Randomized Clinical Trial,” JAMA Network Open 6, no. 8 (2023): e2326463. 37526937 10.1001/jamanetworkopen.2023.26463 PMC10394582

[70] J. Schneider , K. Schlüter , F. Rosenberger , and J. Wiskemann , “Are Percentages of the One‐Repetition Maximum (1‐RM) Suitable for Prescribing Resistance Exercise in Cancer Survivors?—Comparability and Prediction Accuracy of Frequently Used 1‐RM Testing Procedures,” accessed May 3, 2026, 10.21203/rs.3.rs-2165112/v1.

[71] D. Riebe , J. K. Ehrman , G. Liguori , and M. Magal , eds., ACSM's Guidelines for Exercise Testing and Prescription, 10th ed. (Philadelphia, Baltimore, New York, 2018).

