# Supplementary material for: Haematological and Oncological Training Therapy With Stationary Strength and Cardio Machines (HOT) in Routine Cancer Care: A 3‐Year Real‐World Evaluation of Acceptance, Feasibility, Safety, and Effects
Source: Cancer Med. 2026 Jun 12;15(6):e72013. doi: 10.1002/cam4.72013 (PMC13263543; doi:10.1002/cam4.72013)
Supplement: Supplementary file 1 — Supplement 1: Live feedback on movement execution. [file CAM4-15-e72013-s002.pdf]

Supplement 1. Live feedback on movement execution

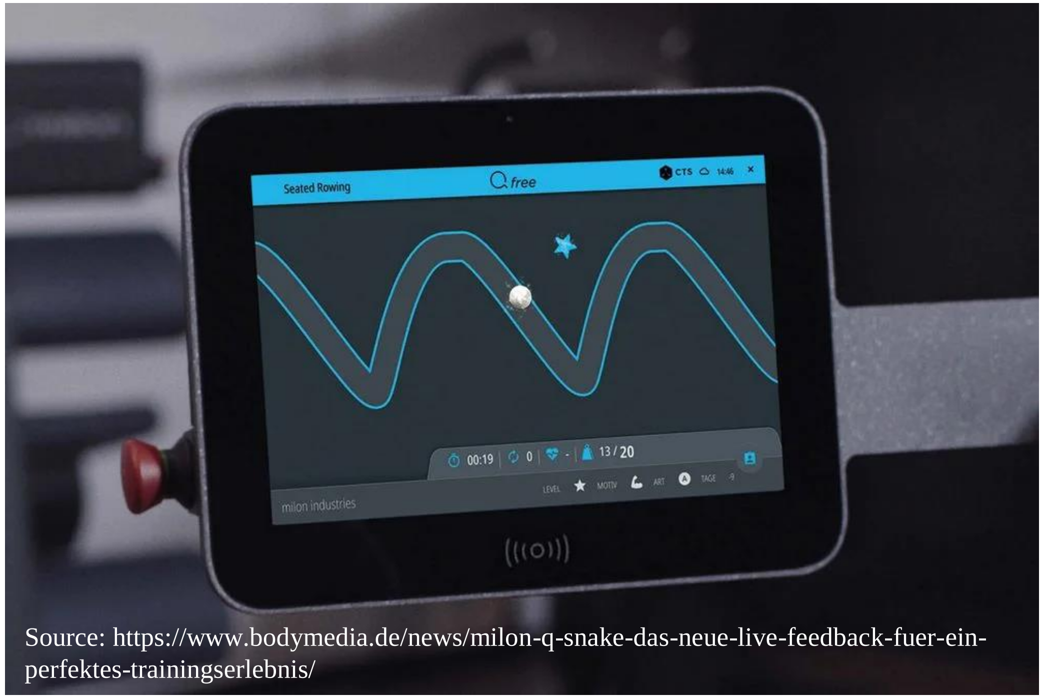

Source: https://www.bodymedia.de/news/milon-q-snake-das-neue-live-feedback-fuer-ein-perfektes-trainingserlebnis/

The device sets the speed of movement in the form of a snake. The aim is to keep the ball in the snake, e.g. leg extension = ball rises, leg flexion = ball sinks. If performed correctly, a star appears as a reward.
The monitor also shows the remaining exercise time, the number of repetitions already completed, and the weight.
