# Supplementary material for: Haematological and Oncological Training Therapy With Stationary Strength and Cardio Machines (HOT) in Routine Cancer Care: A 3‐Year Real‐World Evaluation of Acceptance, Feasibility, Safety, and Effects
Source: Cancer Med. 2026 Jun 12;15(6):e72013. doi: 10.1002/cam4.72013 (PMC13263543; doi:10.1002/cam4.72013)
Supplement: Supplementary file 2 — Supplement 2: Procedures for Haematological and Oncological Training Therapy using stationary strength and cardio machines (HOT). [file CAM4-15-e72013-s005.pdf]

Supplement 2. Procedures for Hematological and Oncological Training Therapy using stationary strength and cardio machines (HOT)

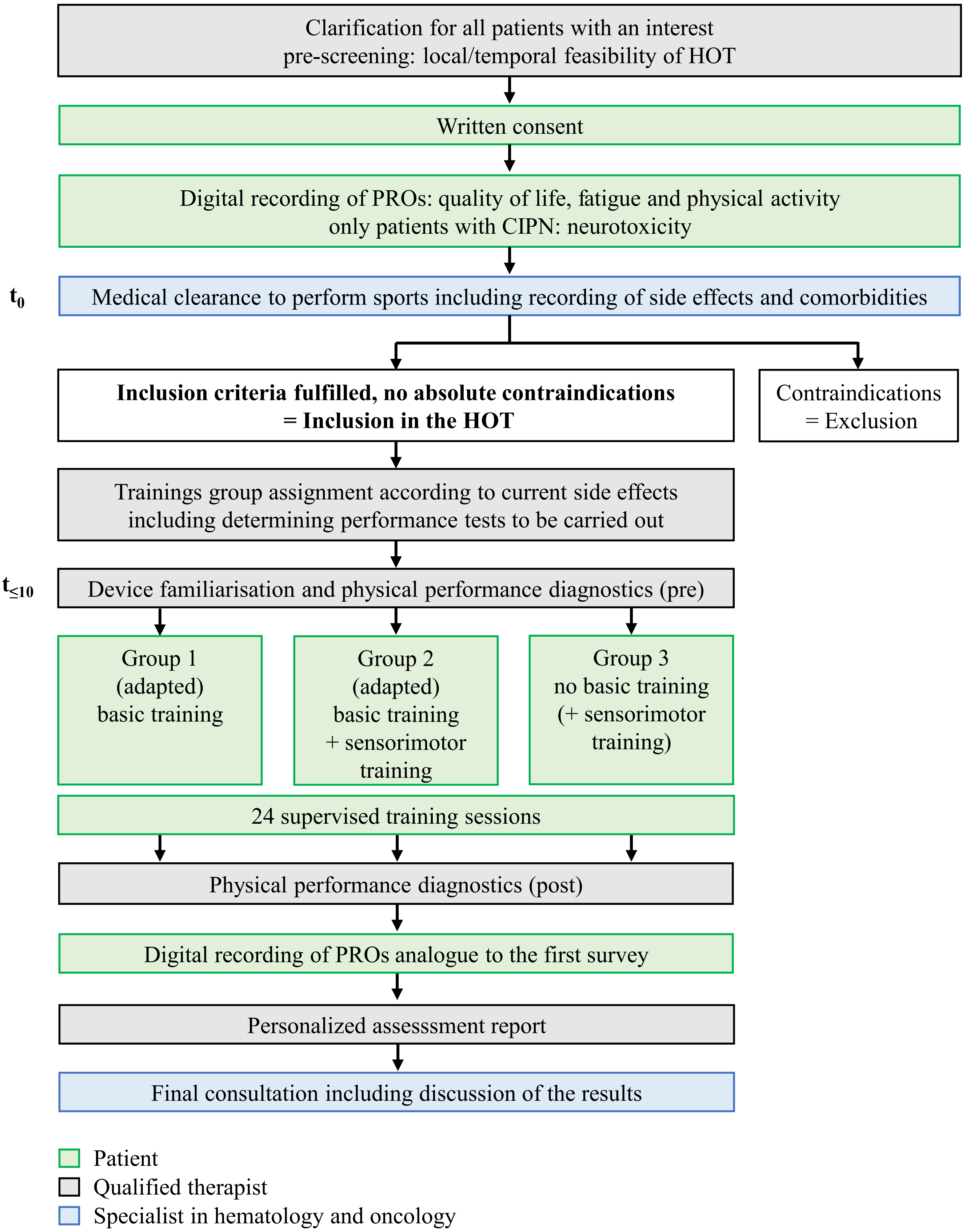

Abbreviations: PROs, patient report outcomes; CIPN, chemotherapy-induced peripheral neuropathy; t_0, day of the medical clearance: $t_{\leq 10}$, pre-performance diagnostics within the first 10 days after the medical clearance
