# Supplementary material for: Haematological and Oncological Training Therapy With Stationary Strength and Cardio Machines (HOT) in Routine Cancer Care: A 3‐Year Real‐World Evaluation of Acceptance, Feasibility, Safety, and Effects
Source: Cancer Med. 2026 Jun 12;15(6):e72013. doi: 10.1002/cam4.72013 (PMC13263543; doi:10.1002/cam4.72013)
Supplement: Supplementary file 3 — Supplement 3: Characteristics of completers versus non‐completers. [file CAM4-15-e72013-s001.pdf]

Supplement 3. Characteristics of completers vs. non-completers

Parameter		Completers	Non-completers	*p*-value
Sample size		53 (76)	17 (24)	
Gender	Women	33 (79)	9 (21)	0.574
	Men	20 (71)	8 (29)	
Age [years]		63 ± 11	65 ± 13	0.387
Professional status	Retired	30 (75)	10 (25)	0.663
	Working	20 (77)	6 (23)	
	Unemploymed	1 (50)	1 (50)	
Sports activities before diagnosis	Continuously active	27 (77)	8 (23)	0.872
	In individual stages of life	22 (73)	8 (27)	
	School sports only	3 (75)	1 (25)	
Place of treatment/aftercare	University Medical Center	21 (78)	6 (22)	0.784
	Practicing physician	32 (74)	11 (26)	
Type of cancer	Solid tumours	33 (77)	10 (23)	1.000
	Haematological neoplasms	20 (74)	7 (26)	
Entity	Lymphomas incl. CLL	13 (68)	6 (32)	0.241
	Gynecological/breast tumours	13 (76)	4 (24)	
	Gastrointestinal tumours	11 (85)	2 (15)	
	Myeloproliferative diseases	6 (86)	1 (14)	
	Urogenital tumours	5 (100)	0 (0)	
	Neuro-oncological tumours	0 (0)	2 (100)	
	Other	5 (71)	2 (29)	
Therapy intention	Curative	29 (81)	7 (19)	0.408
	Palliative	24 (71)	10 (29)	
Therapy phase at inclusion	Undergoing treatment	32 (80)	8 (20)	0.174
	Follow-up care	19 (76)	6 (24)	
	Watch and wait	2 (50)	2 (50)	
	Therapy break	0 (0)	1 (100)	
Comorbidities	Cardiovascular diseases	21 (72)	8 (28)	0.778
	Diabetes mellitus	7 (70)	3 (30)	0.699
	Osteoporosis	2 (40)	3 (60)	0.088
Quality of life and symptoms at the time of inclusion	Global QoL[a]	50 (42, 67)	58 (33, 67)	0.871
	Total Fatigue Score[b]	58 (44, 69)	55 (46, 71)	0.964
	Neuropathy[c]	31 (26, 37)	43 (36, 49)	0.003*

Data are presented as the number of participants (%) for categorical variables and as mean ± standard deviation or in case of non-normality, as median (Q1, Q3) for continuous variables. Mean differences for continuous variables were tested using independent t test or in case of non-normality, Mann–Whitney U test and chi-squared test or Fisher’s exact test for categorical variables.

[a] According to Quality of Life questionnaire of cancer patients of European Organization for Research and Treatment of Cancer (EORTC-QLQ-C30)

[b] According to Multidimensional Fatigue Inventory (MFI-20)

[c] According to Functional Assessment of Cancer Therapy/Gynecologic Oncology Group - Neurotoxicity 13 Item Version (FACT/GOG-NTX-13), completers: n = 24, non-completer: n = 5

* $p \leq 0.05$
