# Supplementary material for: Haematological and Oncological Training Therapy With Stationary Strength and Cardio Machines (HOT) in Routine Cancer Care: A 3‐Year Real‐World Evaluation of Acceptance, Feasibility, Safety, and Effects
Source: Cancer Med. 2026 Jun 12;15(6):e72013. doi: 10.1002/cam4.72013 (PMC13263543; doi:10.1002/cam4.72013)
Supplement: Supplementary file 4 — Supplement 4: Percentage change in (a) 1‐repetition maximum in the six strengthening exercises of the basic training, (b) relative maximum power on the cycle ergometer, and (c) skeletal muscle mass after completion Hematological and Oncological Training Therapy using stationary strength and cardio machines. [file CAM4-15-e72013-s004.pdf]

**Supplement 4**. Percentage change in (a) 1-repetition maximum in the six strengthening exercises of the basic training, (b) relative maximum power on the cycle ergometer, and (c) skeletal muscle mass after completion Hematological and Oncological Training Therapy using stationary strength and cardio machines

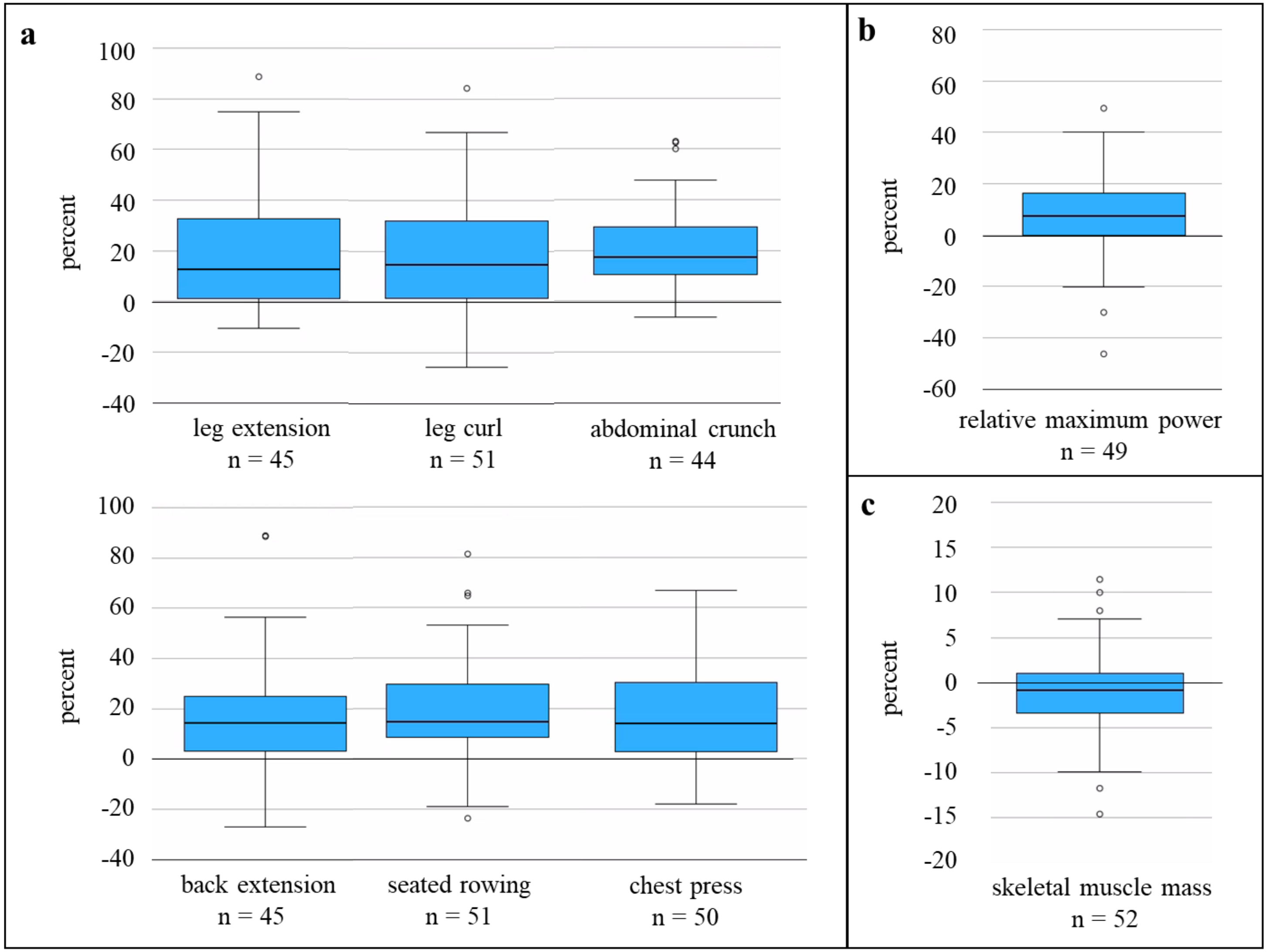


In boxplots, horizontal lines represent the median, boxes quartiles (Q1, Q3) and whiskers extend from Q1 and Q3 to the minimum and maximum values within 1.5 times the interquartile range. The points outside the whiskers are outliers.
